# Design, Implementation, and Evaluation of a School Insecticide-Treated Net Distribution Program in Cross River State, Nigeria

**DOI:** 10.9745/GHSP-D-17-00350

**Published:** 2018-06-27

**Authors:** Angela Acosta, Emmanuel Obi, Richmond Ato Selby, Iyam Ugot, Matthew Lynch, Mark Maire, Kassahun Belay, Abidemi Okechukwu, Uwem Inyang, Jessica Kafuko, George Greer, Lilia Gerberg, Megan Fotheringham, Hannah Koenker, Albert Kilian

**Affiliations:** a Johns Hopkins Center for Communication Programs, Baltimore, MD, USA.; b Tropical Health, LLP, Montagut, Spain.; c Malaria Consortium, Abuja, Nigeria.; d Malaria Consortium, Kampala, Uganda.; e Office of the Governor, Cross River State, Nigeria.; f Division of Parasitic Diseases and Malaria, Center for Global Health, Centers for Disease Control and Prevention, Atlanta, GA, USA.; g U.S. President's Malaria Initiative, U.S. Agency for International Development, Abuja, Nigeria.; h U.S. President's Malaria Initiative, U.S. Agency for International Development, Dar es Salaam, Tanzania.; i U.S. President's Malaria Initiative, U.S. Agency for International Development, Bureau for Global Health, Office of Health, Infectious Disease & Nutrition, Washington, DC, USA.

## Abstract

**Background:**

In 2013, the World Health Organization recommended distribution through schools, health facilities, community health workers, and mass campaigns to maintain coverage with insecticide-treated nets (ITNs). We piloted school distribution in 3 local government areas (LGAs) of Cross River State, Nigeria.

**Methods:**

From January to March 2011, all 3 study sites participated in a mass ITN campaign. Baseline data were collected in June 2012 (N=753 households) and school distribution began afterward. One ITN per student was distributed to 4 grades once a year in public schools. Obubra LGA distributed ITNs in 2012, 2013, and 2014 and Ogoja LGA in 2013 and 2014 while Ikom LGA served as a comparison site. Pregnant women in all sites were eligible to receive ITNs through standard antenatal care (ANC). Endline survey data (N=1,450 households) were collected in March 2014. Data on ITN ownership, population access to an ITN, and ITN use were gathered and analyzed. Statistical analysis used contingency tables and chi-squared tests for univariate analysis, and a concentration index was calculated to assess equity in ITN ownership.

**Results:**

Between baseline and endline, household ownership of at least 1 ITN increased in the intervention sites, from 50% (95% confidence interval [CI]: 44.7, 54.3) to 76% (95% CI: 71.2, 81.0) in Ogoja and from 51% (95% CI: 35.3, 66.7) to 78% (95% CI: 71.5, 83.1) in Obubra, as did population access to ITN, from 36% (95% CI: 32.0, 39.5) to 53% (95% CI: 48.0, 58.0) in Ogoja and from 34% (95% CI: 23.2, 45.6) to 55% in Obubra (95% CI: 48.4, 60.9). In contrast, ITN ownership declined in the comparison site, from 64% (95% CI: 56.4, 70.8) to 43% (95% CI: 37.4, 49.4), as did population ITN access, from 47% (95% CI: 40.0, 53.7) to 26% (95% CI: 21.9, 29.9). Ownership of school ITNs was nearly as equitable (concentration index 0.06 [95% CI: 0.02, 0.11]) as for campaign ITNs (−0.03 [95% CI: −0.08, 0.02]), and there was no significant oversupply or undersupply among households with ITNs. Schools were the most common source of ITNs at endline and very few households (<2%) had nets from both school and ANC.

**Conclusion:**

ITN distribution through schools and ANC provide complementary reach and can play an effective role in achieving and maintaining universal coverage. More research is needed to evaluate the cost-effectiveness of such continuous distribution channels in combination with, or as a potential replacement for, subsequent mass campaigns.

## Abstract (precis)

Three years following a mass bed net distribution campaign, the addition of school-based distribution to antenatal care (ANC) distribution in Cross River State, Nigeria, increased household ownership of any net to nearly 80%, whereas ownership in the comparison area was below 50%. School distribution was nearly equitable among rich and poor, and very few households obtained nets from both ANC and schools, suggesting complementary reach.

## BACKGROUND

Insecticide-treated nets (ITNs) are an effective means of preventing malaria. Over the past 10 years, hundreds of millions of ITNs have been distributed throughout sub-Saharan Africa.^1^ Most of these have been through either targeted or universal mass campaigns, which have been found to raise coverage rapidly and equitably.^2^^–^^5^ However, maintaining these gains can be a challenge. Household ITN ownership and population access to ITNs start to decrease immediately after mass campaigns due to births, migration, and net loss (through repurposing of or damage to the nets). In response, countries have used mass campaigns to replenish ITN coverage every few years. These “top-up” and repeated universal coverage campaigns can be challenging and costly, given the burden of conducting registration visits to every household and the potential for oversupply. Moreover, most households cannot obtain ITNs between mass campaigns.^6^^,^^7^ Although antenatal care (ANC) clinics, the Expanded Program on Immunization (EPI), and retailers also distribute or sell ITNs, the volumes are too low to maintain universal coverage.^8^

In 2013, the World Health Organization's (WHO's) Malaria Policy Advisory Committee recommended the combined use of mass campaigns and continuous distribution channels to maintain universal coverage. Universal coverage is defined as universal access to and use of ITNs by populations at risk of malaria and is usually interpreted as the broad goal of distributing 1 net for every 2 people.^9^^,^^10^ Examples of continuous distribution channels include ANC and EPI as well as community-based platforms, religious networks, agricultural and food-security support schemes, the private and commercial sector, and schools.^11^

WHO recommends the combined use of mass bed net campaigns and continuous distribution to maintain universal coverage.

Schools have long been used as platforms for public health interventions related to nutrition, personal and environmental hygiene, deworming, vaccination, and malaria treatment and surveillance.^12^^–^^16^ While the primary target beneficiaries for school distribution are household members, students can serve as conduits to households. They can transport ITNs from school to home, where household members can allocate the ITN as needed. Students can also share messages on the importance of using nets with household members.^16^

Schools are a promising channel for ITN distribution for several reasons. First, many countries have high rates of school enrollment, particularly at the primary school level.^17^ Second, schools' reach into communities is often as good as, if not better than, the reach of the health sector; in many cases, schools outnumber health facilities in the same area. Third, the number of grades receiving ITNs can be increased or decreased based on the number of ITNs required to maintain desired coverage levels.^18^ This level of flexibility and reach is not possible with ANC and EPI distribution channels. Fourth, schools have existing structures that make ITN distribution feasible. For example, they have student registers, eliminating the time and costs of household registration visits. They also have lockable storage areas that can temporarily store ITNs. Furthermore, teachers are literate and numerate personnel who can complete basic reports and pass information on to students. Teachers may also value preventive health behaviors and care deeply about students' health and its implications on absenteeism and education outcomes.

Schools are a promising channel for bed net distribution.

Because of these advantages, several countries (Ghana, Nigeria, Senegal, Tanzania, Uganda, Zambia, and Zimbabwe) have piloted school-based distribution while several others have included it in their national malaria strategic plans. However, there is little published evidence on the impact of school distribution of ITNs. This article reports on a proof-of-principle study to assess whether adding the distribution of ITNs to a few school grades to the existing ANC channel could sustain household ownership of at least 1 ITN and population ITN access 3 years after a mass campaign in Cross River State, Nigeria.

## METHODS

### Setting

This study was conducted in Cross River State in the South-South zone of Nigeria, a region that is highly endemic for malaria. In consultation with the State Ministries of Education and Health, we selected Obubra, Ogoja, and Ikom local government areas (LGAs) as they have similar populations and are equally accessible. The populations for Obubra, Ogoja, and Ikom were projected to be 205,000, 204,000, and 193,000 respectively, in 2012 based on the 2006 national census and an estimated growth rate of 3.2%.^19^

### Study Design

**FIGURE 1:**
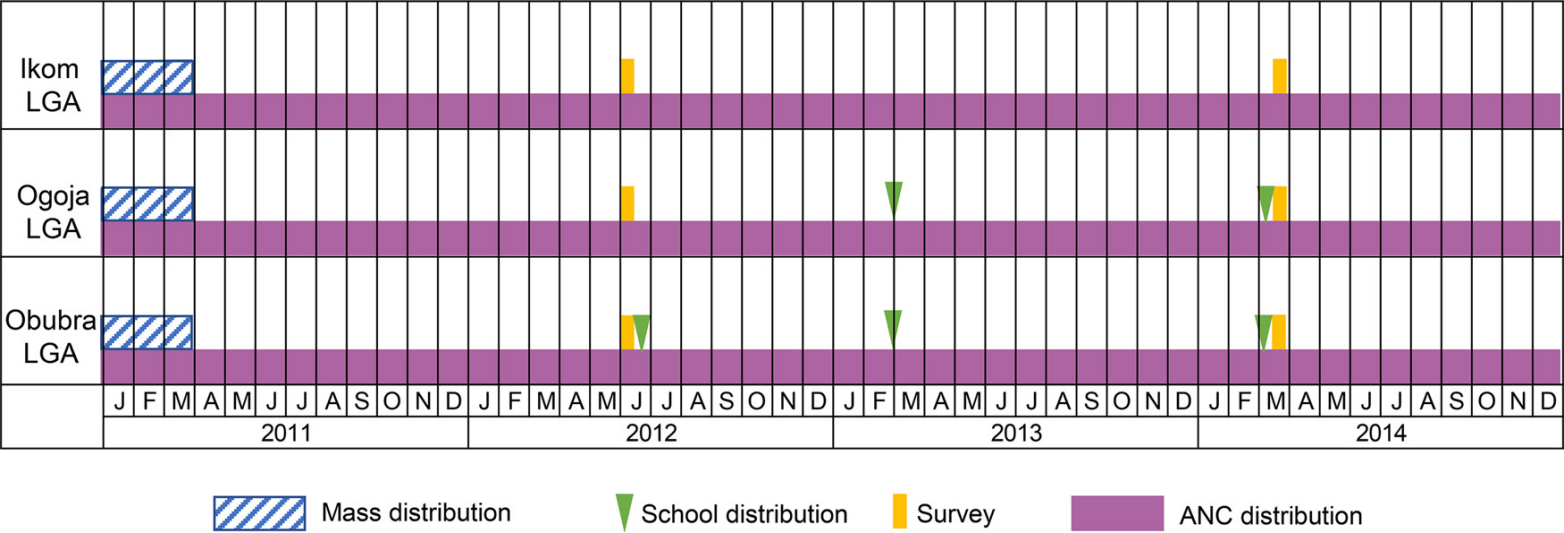
Modes and Timing of ITN Distribution and Baseline and Endline Surveys, Cross River State, Nigeria, 2011–2014 Abbreviations: ANC, antenatal care; ITN, insecticide-treated net; LGA, local government area.

This study was a before-after assessment of intervention areas with a comparison area using cross-sectional household surveys. Cross River State distributed nets to all 3 study sites during the first wave of the mass ITN campaign, which ran from January to March 2011. The study sites did not participate in the second wave of the mass ITN campaign, which issued nets to several other LGAs in Cross River State from October 2011 to February 2012. We collected baseline data in June 2012 (peak of the rainy season; 15 months after the mass campaign) and implemented the first school distribution in Obubra LGA immediately afterward. During these distributions, schools distributed nets to students in 4 grades during a 1-week period. In late February 2013 and March 2014, nets were distributed in both Obubra and Ogoja LGAs. The endline surveys were fielded a few days after the last distribution in March 2014 (end of the dry season). Ikom LGA served as a comparison site and did not receive nets for school distribution. However, all 3 sites received ITNs for distribution to pregnant women at their first ANC visit as standard practice in Cross River State (Figure 1).

**FIGURE 2:**
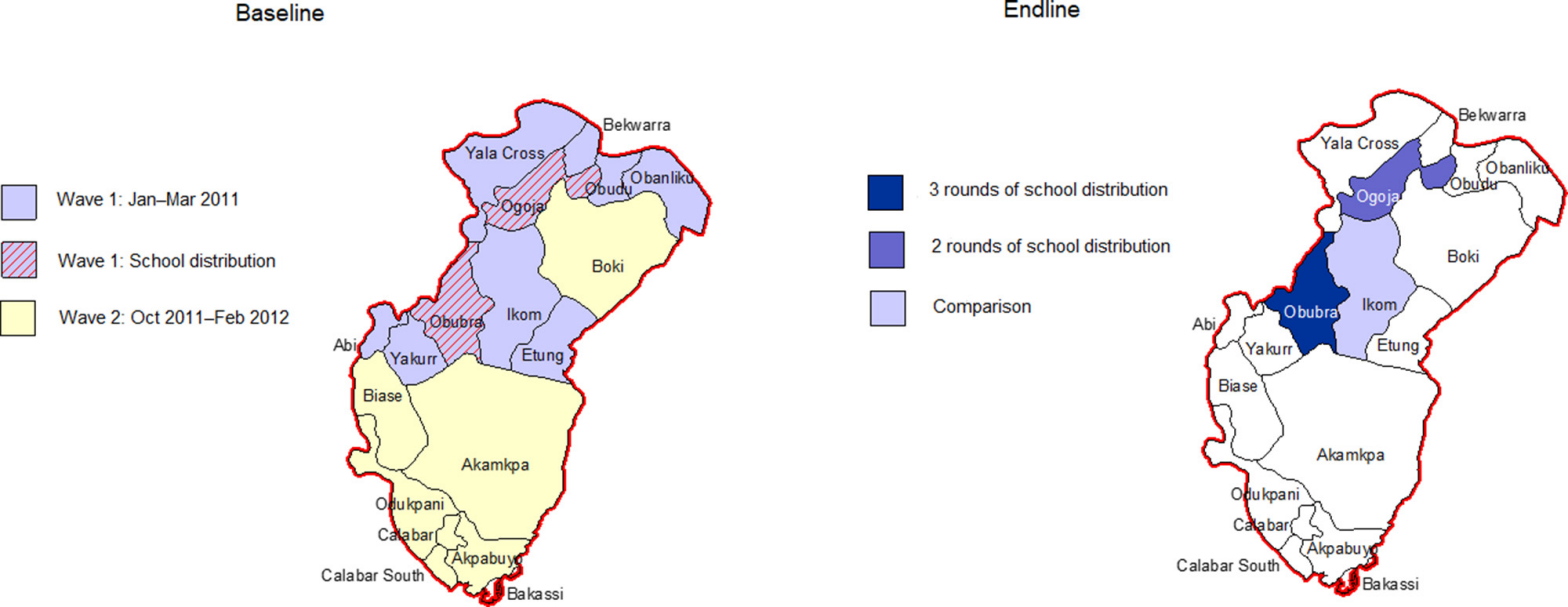
Maps of Baseline and Endline Survey Strata by Mode of ITN Distribution per LGA, Cross River State, Nigeria Abbreviations: ITN, insecticide-treated net; LGA, local government area.

The baseline also served as an evaluation of the statewide mass campaign, while the endline was used only to evaluate the continuous (school + ANC) distribution program. For these reasons, the sample size and stratification approach differed between baseline and endline. The 2 primary strata for the baseline survey were the 2 waves of the mass campaign, and the 2 school-distribution LGAs were oversampled as part of the wave 1 stratum (Figure 2, left panel). The baseline survey covered the whole state with a total of 75 clusters across 10 LGAs; 45 for areas covered by the first wave of the mass campaign (15 for Obubra LGA, 15 for Ogoja LGA, and 15 for the rest of wave 1), and 30 clusters for the second wave (the remaining LGAs). In contrast, the endline survey was limited to the school distribution pilot area (Figure 2, right panel) with a total of 90 clusters, 30 in each of the 3 strata across the 3 LGAs. Because the baseline survey covered a larger group of LGAs than the endline, we ran a sensitivity analysis to assess the comparability between the endline and baseline samples.

Our target sample size for the school baseline and endline assessment was 765 and 1,530 households, respectively. The target sample size of the endline survey was calculated to detect a 12 percentage-point difference in ITN coverage between implementation and comparison sites assuming 5% non-response, a design effect of 1.75, power of 80%, and a 95% confidence interval (CI) for the 2-sided assessment.

The baseline and endline surveys used a stratified, multistage cluster sampling design. First, using population data from the 2006 census, 75 (baseline) and 90 (endline) wards were selected using probability proportionate to size. Thereafter, 1 community (settlement) per ward was selected using simple random sampling from a complete list of settlements for that ward, and served as the cluster. Within each cluster, all households were eligible for selection. A list of households was prepared by the survey team on the day of the survey and the households for interview were selected using simple random number lists. If a cluster had more than 200 households, an equal-size section approach was used and 1 section was randomly chosen from the household list. Households were defined as “people eating from the same pot,” which was the definition used in the mass campaign. The target for household-level interviews was the head of household or his/her spouse.

The questionnaire was based on the 2010 *Malaria Indicator Survey* and focused on household ownership, access to, and use of ITNs.^20^ Additional questions were added to capture several processes specific to the school distribution such as number of ITNs received through school and sources of information about net use or hanging.

### Program Description

#### Design, Coordination, and Planning

We used the population-based NetCALC modeling tool to estimate the number of nets and grades needed for the pilot.^21^ The model assumed that at least 80% of households would own at least 1 ITN after the mass campaign (Supplement). Input data included demographic characteristics from the 2006 census and ANC and school attendance rates from the 2008 Demographic and Health Survey (a gross attendance ratio of 110% was used for primary schools and 95% for secondary schools as well drop-out rates of 20% and 25%, respectively).^22^

We met jointly with health and education officials at the state and LGA levels to coordinate planning. Together, we conducted field visits and in-depth interviews with teachers and head teachers to develop the guidelines. Samples of implementation materials from the pilot can be found at www.continuousdistribution.org.

Stakeholders preferred the month of March for school distribution for several reasons. First, it was before the high malaria transmission (rainy) season. Second, it was relatively soon (within 1 year) after the mass distribution in those LGAs, preventing a prolonged gap between distributions. Finally, it did not conflict with school exams. They chose grade levels that were 1 to 3 years apart to ensure most households with children could receive at least 1 ITN every 2 to 3 years: primary year 1, primary year 4, junior secondary school year 1 (7th year of education), and senior secondary school year 1 (10th year of education). Heads of schools and teachers of the selected grades also received ITNs as incentives for participation. Only public schools were included in the pilot.

#### ITN Quantification

We used second-term attendance numbers from school records to calculate the number of ITNs required. The second term was chosen because attendance usually stabilizes by this time. A buffer stock was considered unnecessary.

Instead of conducting household registration visits, school attendance records were used to calculate the number of ITNs required.

#### Training and Microplanning

A cascade training model was used. Heads of schools traveled to the LGA level for an orientation on distribution, completion of forms, supervision, and social and behavior change communication (SBCC) messages. Heads of schools then returned to their schools and trained their teachers. During trainings, heads of schools brought enrollment data, which we used to allocate nets to schools and grades. Microplans for ITN transport and storage were also created during these workshops.

#### ITN Transport and Storage

We hired a private transport company to deliver ITNs from the state warehouse (to which they were directly shipped after arrival at port) to the LGA warehouse once school microplanning data were verified, and then from the LGA warehouse to schools. Nets were in storage for a minimal amount of time to reduce the potential for leakage; they arrived at the LGA warehouse 3 days before distribution and at the school 1 to 2 days before distribution. Schools kept ITNs in store rooms or in the head of school's office.

#### ITN Distribution

One ITN per student was distributed to 4 grades once a year in public schools. Nets were distributed during the school break (30–45 minutes) to minimize disruption. Teachers in target grades first taught students about the benefits of ITN use and net maintenance, then each student was called forward to receive an ITN and sign the register. Students received Olyset or DawaPlus 2.0 ITNs. Teachers opened the packaging before handing ITNs over to discourage resale. Since there were concerns about other people taking nets from primary school 1 students, schools asked their parents or their representatives to come and pick up the net on the child's behalf. A total of 50,138 ITNs were distributed during the pilot; 8,444 in Obubra LGA in 2012; 20,545 in Obubra and Ogoja in 2013, and 21,149 in Obubra and Ogoja in 2014.

Each year, we distributed 1 ITN per student to 4 grades, which were 1 to 3 years apart to ensure most households with children could receive at least 1 ITN every 2 to 3 years.

#### Supervision

Heads of schools supervised teachers as ITNs were distributed. In addition, pairs of external monitors, composed of state and LGA health and education officials, NGO representatives, and local consultants, visited selected schools on distribution days. Supervisors sought to ensure that ITNs were distributed to the right grades and that registered students were in the classroom, received education on malaria prevention, received an ITN, and signed the register.

#### Social and Behavior Change Communication

New SBCC activities were added each year and they were implemented only in Obubra and Ogoja shortly before, during, and after each school distribution period. In 2012, stakeholder meetings were held with ward, LGA, and community opinion leaders. The school distribution program was also discussed at parent-teacher association meetings and in class by teachers. Key messages included who is eligible to get a net, how to obtain a net, how to use the net, and how to care for it. In 2013, a teacher's guide was developed, containing key messages and suggestions for classroom and assembly activities. In 2014, teachers used additional materials, including a poster, a comic strip, and a malaria protection pledge.

#### Monitoring

Waybills and stock cards were used to track the flow of ITNs from the state to the school level. Each school had an allocation list and a distribution register. One head of school collected forms from other schools in his or her ward and handed them over to the LGA education executive officer.

### Data Collection and Analysis

The study goal was to assess household ITN ownership, access, use, and equity in each of the 3 sites over time by using definitions from the Roll Back Malaria Monitoring and Evaluation Reference Group (MERG).^23^ Key indicators included:

- ITN ownership (proportion of households that owned at least 1 ITN)
- Proportion of households with at least 1 ITN for every 2 people
- Proportion of the population with access to an ITN within their household (the proportion of the population that could be protected by an ITN, assuming that each ITN in a household can be used by 2 people)
- Net use (the percentage of a given population group that slept under an ITN the night before the survey)
- Equity (access to any ITNs across economic quintiles)

A question on the source of each net was added to the ITN roster at endline to assess the contribution of each channel and the degree of overlap in the reach of the channels. Answer options included mass campaign, ANC, health facility, community drug distributors, schools, mosque or church, pharmacy, shop or supermarket, market, hawker, school, and other. Respondents were asked if they had a child in the selected grades during each of the last 3 years and if the household had received a net from school or from ANC.

Data were entered using EpiData 3.1 software (EpiData Association, Odense, Denmark) with double entry and record validation. Cleaned data sets were then transferred to Stata 13.1 software package (Stata Corp., College Station, Texas, USA) for further consistency checks and cleaning before data processing and analysis. Sampling weights (inverse of the probability of cluster and household selection) were used to reflect the unbalanced sampling strategy for the baseline survey. In addition, all analyses for baseline and endline survey accounted for the cluster survey design by using the appropriate commands in the statistical software package.

A wealth index was computed at the household level using principal component analysis (PCA), using variables for household amenities, assets, livestock, and other characteristics that are related to a household's socioeconomic status. Quintiles were calculated separately for each stratum. Lorenz concentration curves were produced by plotting the cumulative distribution of wealth quintiles among households with the outcome of interest (e.g., ITN ownership from school distribution) against the respective distribution among all sampled households as described by O'Donnell et al.^24^ A concentration index was used to analyze outcome differences by wealth quintile. Standard errors and confidence intervals for the concentration indices were calculated using the formula suggested by Kakwani et al.^23^

Data on sources of ITN information were obtained by asking respondents if they had heard or seen any messages about net hanging or use in the past 6 months, and if so, where they had seen or heard the message and what types of messages were recalled. The latter two were then summarized as mean number of sources of information and mean number of messages recalled. Additional questions included whether the respondent had discussed net use with family members, and whether he or she intended to use a net every night. Respondents from households with children in school were asked if they knew whether the child learned about malaria or ITNs at school.

Statistical analysis used contingency tables and chi-squared tests for univariate analysis. A difference-in-difference approach was also used to assess the treatment effect between intervention and comparison groups over time. Statistical significance was defined at the *P*<.05 level.

### Ethical Clearance

Ethical clearance was obtained for conducting human subject research from the Johns Hopkins Bloomberg School of Public Health Institutional Review Board (IRB #4073 baseline; #5553 endline), as well as from the National Health Research Ethics Committee of Nigeria. All participants provided informed consent.

## RESULTS

This study ran from 2012 to 2014 in Cross River State, Nigeria. Obubra LGA implemented its first round of school ITN distribution in mid-2012, 15 months after the mass campaign, and then again in March 2013 and March 2014. Ogoja LGA implemented its first round in March 2013, 23 months after the mass campaign, and then again in March 2014. Ikom LGA served as a comparison area. The quality of implementation was good since almost all (98%) targeted school children received an ITN.

The final sample size obtained for analysis for the baseline survey was 753 households (98% of the target), with 502 households in the 2 school implementation groups and 251 in the comparison group. The sample obtained for the endline evaluation was 1,450 (95% of the target) with rates of 94%, 96%, and 95%, respectively, for Obubra, Ogoja, and Ikom LGAs. At the population level, the baseline survey included 3,593 de-facto household members, of which 96% were usual household members (de-jure population). For the endline survey, the de-facto population was 8,186, of which 97% were de-jure.

### Household Characteristics at Baseline and Endline

Key demographic characteristics at baseline are shown in Table 1. With a few exceptions, neither of the 2 school distribution LGAs differed significantly from the surrounding LGAs in the first wave of the ITN mass distribution campaign. Household demographics and access to safe drinking water and latrines were similar in all sites and so was the main type of house construction. A lower percentage of the heads of households in Obubra LGA was literate than the other LGAs, a lower percentage had a secondary education, and a significantly lower percentage owned a radio or mobile phone, suggesting that this LGA was overall socioeconomically somewhat worse off than the rest. Interestingly, ownership of a means of transport was similar between the 2 school distribution LGAs but higher in the comparison group. Although the baseline survey did not have a large enough sample for the comparison LGA (Ikom) to allow precise estimates of key household characteristics, the results from this LGA alone did not suggest a deviation from the average of the LGAs in the rest of wave 1 (data not shown).

**TABLE 1.** Baseline Characteristics of Survey Households, by Comparison^a^ and School-Based Distribution Intervention Sites (N=753)

	Comparison	Intervention		*P* Value
	Rest of Wave 1 (N=251)	Ogoja LGA: 2 Rounds (N=286)	Obubra LGA: 3 Rounds (N=216)	
No. of de-jure household members, mean	4.7	4.5	4.9	.33
No. of persons per sleeping room	2.2	2.4	2.3	.07
Households with any children under 5, %	33.1%	38.5%	37.1%	.56
Households with a pregnant woman, %	8.6%	9.6%	12.3%	.51
Households with any eligible school children, %	–	36.7%	42.8%	.19^b^
Age of head of household, years, mean	41.3	43.4	41.4	.24
Female-headed households, %	18.4%	23.5%	22.5%	.57
Educational achievement of head of household, %				.09
Non-literate	10.7%	11.8%	21.4%	
Primary	22.6%	22.1%	31.3%	
Secondary	48.5%	48.2%	29.3%	
Tertiary	18.1%	18.0%	18.1%	
Household access to safe water, %	41.2%	35.8%	23.6%	.47
Household access to any latrine, %	72.0%	66.0%	59.8%	.33
Houses with modern roof (e.g., sheets, tiles), %	89.7%	92.2%	91.2%	.81
Household ownership of radio, %	86.4%	81.8%	68.9%	.02
Household ownership of mobile phone, %	83.7%	80.3%	66.7%	.06
Household ownership of any means of transport, %	73.8%	58.2%	55.7%	.007
Households registered by ITN campaign, %	48.0%	37.8%	44.9%	.36
Household received any net from campaign, %	65.8%	47.0%	47.6%	.006
No. of ITNs received, if any, mean	1.88	1.73	1.83	.61

Abbreviations: ITN, insecticide-treated net; LGA, local government area.

a The rest of the LGAs (8 total) in the wave 1 distribution served as the comparison group at baseline.

b Comparing Ogoja to Obubra LGA.

Registration rates for the mass campaign were not very high (range, 37.8% to 48.0%) but did not differ significantly between sites (Table 1). However, in the baseline comparison group, more households received ITNs from the campaign compared with the intervention LGAs; as a result the overall reach of the campaign was somewhat higher in the comparison group. Among households that received nets, the number of nets per household was similar across all sites.

Because the baseline comparison group comprised 8 LGAs (Ikom, Etung, Yakurr, Abi, Obanliku, Obudu, Bekwarra, and Yala Cross) and the comparison group at endline sampled in Ikom LGA only, further analyses were conducted to evaluate whether the larger group (labeled “Rest of Wave 1” in the tables) was appropriate to use as a comparison area. Table 2 shows that the larger group and Ikom LGA were similar in composition and socioeconomic status. While somewhat fewer households in Ikom LGA were registered for the campaign (32.8% vs. 48.0%, respectively), there were no statistically significant differences in their ability to own an ITN from the campaign or in the number of ITNs received.

**TABLE 2.** Baseline Characteristics for All Non-School Intervention LGAs in the Wave 1 Distribution (Baseline Comparison Group) and Ikom LGA Alone (Endline Comparison Group)

	Rest of Wave 1 (N=251)	Ikom LGA (N=34)	*P* Value
No. of de-jure household members, mean	4.7	4.8	.98
No. of persons per sleeping room	2.2	2.4	.20
Households with any children under 5, %	33.1%	26.0%	.16
Households with a pregnant woman, %	8.6%	7.5%	.86
Households with any eligible school children, %	–	–	
Age of head of household, years, mean	41.3	43.6	.38
Female-headed households, %	18.4%	15.2	.50
Educational achievement of head of household, %			.14
Non-literate	10.7%	13.5%	
Primary	22.6%	16.8%	
Secondary	48.5%	36.2%	
Tertiary	18.1%	33.5%	
Household access to safe water, %	41.2%	28.5%	.31
Household access to any latrine, %	72.0%	50.3%	.06
Houses with modern roof (e.g., sheets, tiles), %	89.7%	96.6%	.37
Household ownership of radio, %	86.4%	82.4%	.38
Household ownership of mobile phone, %	83.7%	89.9%	.44
Household ownership of any means of transport, %	73.8%	69.0%	.50
Households registered by ITN campaign, %	48.0%	32.8%	.02
Household received any net from campaign, %	65.8%	52.2%	.13
No. of ITNs received, if any, mean	1.88	1.88	.94

Abbreviations: ITN, insecticide-treated net; LGA, local government area.

### Household ITN Ownership and Population Access

Household ownership of ITNs before and after the school distribution is presented in Table 3. Improvements in ownership were seen at both intervention sites. In Obubra LGA, the proportion of households with any ITN increased by 27 percentage points (from 51% to 78%) while the proportion of households with enough ITNs to cover all household members (at least 1 ITN for 2 people) increased by 13 percentage points (from 17% to 30%). Interestingly, Ogoja LGA, which had waited 2 years after the mass distribution campaign to start school distribution, experienced similar levels of improvement, with ITN ownership increasing from 50% to 76% and ownership of 1 ITN for 2 people increasing from 18% to 30%. In contrast, rates in the comparison group at endline were significantly lower (*P*<.05) than the 2 school distribution LGAs with only 43% of households owning any ITN and 14% owning at least 1 ITN for 2 people.

**TABLE 3.** ITN Ownership, Access, and Use (%) at Baseline and Endline, by Comparison^a^ and Intervention Sites

	Baseline (N=753)			Endline (N=1,450)		
	Rest of Wave 1(Comparison)	Ogoja LGA(2 Rounds)	Obubra LGA(3 Rounds)	Ikom LGA(Comparison)	Ogoja LGA(2 Rounds)	Obubra LGA(3 Rounds)
**Household level**						
Owns at least 1 ITN	63.9 (56.4, 70.8)	49.5 (44.7, 54.3)	51.1 (35.3, 66.7)	43.3(37.4, 49.4)	76.4(71.2, 81.0)	77.9(71.5, 83.1)
Owns at least 1 ITN per 2 people	24.4 (17.8, 32.5)	17.7 (13.0, 23.7)	17.4 (11.8, 25.0)	13.9(10.7, 17.8)	29.9(25.1, 35.2)	30.3(26.1, 34.8)
**ITN supply**						
Less than 1 ITN per 3 people	21.1(16.2, 27.2)	16.8 (13.1, 21.5)	20.8 (15.1, 27.9)	20.5(15.8, 26.2)	23.2(19.8, 26.9)	23.6(20.5, 27.0)
1 ITN per 3 people	18.4 (14.3, 23.2)	15.0(10.5, 20.9)	12.9(7.8, 20.6)	8.9(6.5, 12.0)	23.4(19.3, 27.9)	24.0(20.3, 28.1)
1 ITN per 2 people	21.1 (16.7, 26.2)	13.5 (9.7, 18.6)	14.5 (9.5, 21.6)	12.2(9.1, 16.2)	23.8(19.6, 28.5)	22.8(19.0, 27.0)
1 ITN or more per person	3.3 (1.2, 9.2)	4.2 (2.3, 7.6)	2.9 (1.1, 7.5)	1.7(0.9, 16.2)	6.2(4.1, 9.2)	7.5(5.2, 10.8)
**Population level**						
Population access to ITN^b^	46.8 (40.0, 53.7)	35.7 (32.0, 39.5)	33.5(23.2, 45.6)	25.7(21.9, 29.9)	53.1(48.0, 58.0)	54.7(48.4, 60.9)
ITN use previous night	41.8 (35.6, 48.3)	28.9 (26.1, 31.8)	28.5(17.9, 42.0)	16.8(13.7, 20.4)	24.0(20.6, 27.7)	31.6(26.1, 37.6)
ITN use previous night among population with access to ITN	92.5 (86.3, 99.0)	84.7 (81.4, 88.5)	88.3(77.9, 99.8)	66.7(63.6, 70.3)	45.7(42.3, 49.4)	61.2(55.3, 66.7)

Abbreviations: ITN, insecticide-treated net; LGA, local government area.

All data shown as % (95% confidence interval).

a The rest of the LGAs (8 total) in the wave 1 distribution served as the comparison group at baseline, while Ikom LGA served as the comparison at endline.

b Proportion of the population with access to an ITN within their household (assuming each ITN in a household can be used by 2 people).

Household ownership of and population access to ITNs increased in the intervention areas after school distribution was implemented.

**FIGURE 3:**
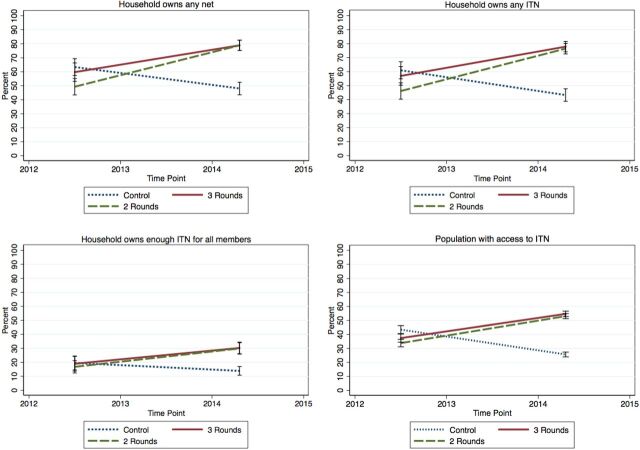
Trends in ITN Indicators From Baseline to Endline Abbreviation: ITN, insecticide-treated net.

Table 3 also shows that there was no major increase of oversupply (1 ITN or more per person) nor of severe undersupply (less than 1 ITN per 3 people) in the intervention LGAs, meaning that gains in coverage were mainly in the “enough” (1 ITN per 2 people) and “almost enough” (1 ITN per 3 people) categories. Similar trends were also seen in population access to an ITN within the household. At endline, population ITN access had increased from 34% to 55% in Obubra LGA and from 36% to 53% in Ogoja LGA. ITN access decreased over time in the comparison areas (from 47% in the baseline comparison group to 26% in Ikom LGA at endline). Trends for ITN indicators are shown in Figure 3.

There was no major increase of oversupply nor of undersupply of ITNs in the intervention areas.

Table 3 also presents ITN use by the general population at baseline and endline. Results clearly show the influence of seasonality on ITN use in this context. While use rates among those who had access to an ITN within the household were generally high at baseline (85% to 93%), which had been done at the peak of the rains, they were much lower at the endline survey (46% to 67%), which was conducted at the end of the dry season. Between the 2 school distribution LGAs, Obubra showed better use rates than Ogoja (61% vs. 46%, respectively; *P*<.05), but Ikom LGA (comparison) had similarly high rates (67%) as Obubra LGA. However, even with the increased “use gap” at endline, overall ITN use was highest in Obubra LGA, followed by Ogoja LGA and Ikom LGA (comparison), and this trend was statistically significant (*P*<.001). There was no indication that ITNs from school distribution were used less or more the previous night than ITNs from other sources both in univariate comparison and multivariate regression analysis (*P*>.05).

The result from the difference-in-difference analysis is shown in Table 4. The changes in ITN coverage between baseline and endline surveys as shown in Table 3 are expressed as a percentage-point difference comparing the intervention sites (3 or 2 rounds of school distributions) against the comparison group. This difference-in-differences can be interpreted as the overall percentage-point gain in ITN coverage compared with the comparison group combining the decrease observed in the comparison areas with the increases in the intervention areas. This result or “treatment effect” is then tested against the hypothesis that there is no difference between intervention and control. As one would expect, the largest gains of 59 percentage-point increases were seen for the indicator “households owning at least 1 ITN” followed by a 47 to 50 percentage-point gain in population access and 28 to 29 percentage-point gain in households with enough ITNs for all members. Consistently, the comparison between the 2 intervention arms showed no difference in impact of 3 versus 2 rounds of distributions. Using the Ikom LGA subset as the comparison group at baseline rather than the rest of wave 1 did not change the magnitude of the effects, and differences were still statistically significant at the .05 level (data not shown).

**TABLE 4.** Difference-in-Difference Analysis on Core ITN Indicators

Comparison	Difference-in-Differences	*P* Value
**HH owns any ITN**		
Obubra (3 rounds) vs. Comparison^a^	58.8%	<.001
Ogoja (2 rounds) vs. Comparison^a^	58.9%	<.001
Obubra (3 rounds) vs. Ogoja (2 rounds)	−0.01%	.99
**HH owns at least 1 ITN per 2 people**		
Obubra (3 rounds) vs. Comparison^a^	28.2%	<.001
Ogoja (2 rounds) vs. Comparison^a^	28.8%	<.001
Obubra (3 rounds) vs. Ogoja (2 rounds)	−0.6%	.91
**Population access to ITN within HH** ^b^		
Obubra (3 rounds) vs. Comparison^a^	49.6%	<.001
Ogoja (2 rounds) vs. Comparison^a^	47.2%	<.001
Obubra (3 rounds) vs. Ogoja (2 rounds)	2.4%	.42

Abbreviations: HH, household; ITN, insecticide-treated net.

a The rest of the LGAs (8 total) in the wave 1 distribution served as the comparison group at baseline, while Ikom LGA served as the comparison group at endline.

b Proportion of the population with access to an ITN within their household (assuming each ITN in a household can be used by 2 people).

### Sources of ITNs

Sources of ITNs at endline for all surveyed households are shown in Table 5. The school channel was the most common source of ITNs at endline, with 44% (Obubra) and 43% (Ogoja) of all surveyed households reporting owning ITNs from the school distribution. In comparison, 29% (Obubra) and 18% (Ogoja) of households owned any campaign net and 9% and 10%, respectively, owned any net from ANC.

The most common source of ITNs in the intervention areas at endline was schools, followed by mass campaigns and ANC.

At baseline, the overall proportion of households that owned any ITNs from the campaign was only slightly higher in the comparison group than the intervention groups, but it was the main source of ITNs at endline for the comparison group. Antenatal care was the least common source of ITNs; only 2.5% of households in Ikom LGA had any ITNs from ANC. There was very little overlap between the continuous distribution channels. Less than 2% of households had an ITN from both ANC and schools.

**FIGURE 4:**
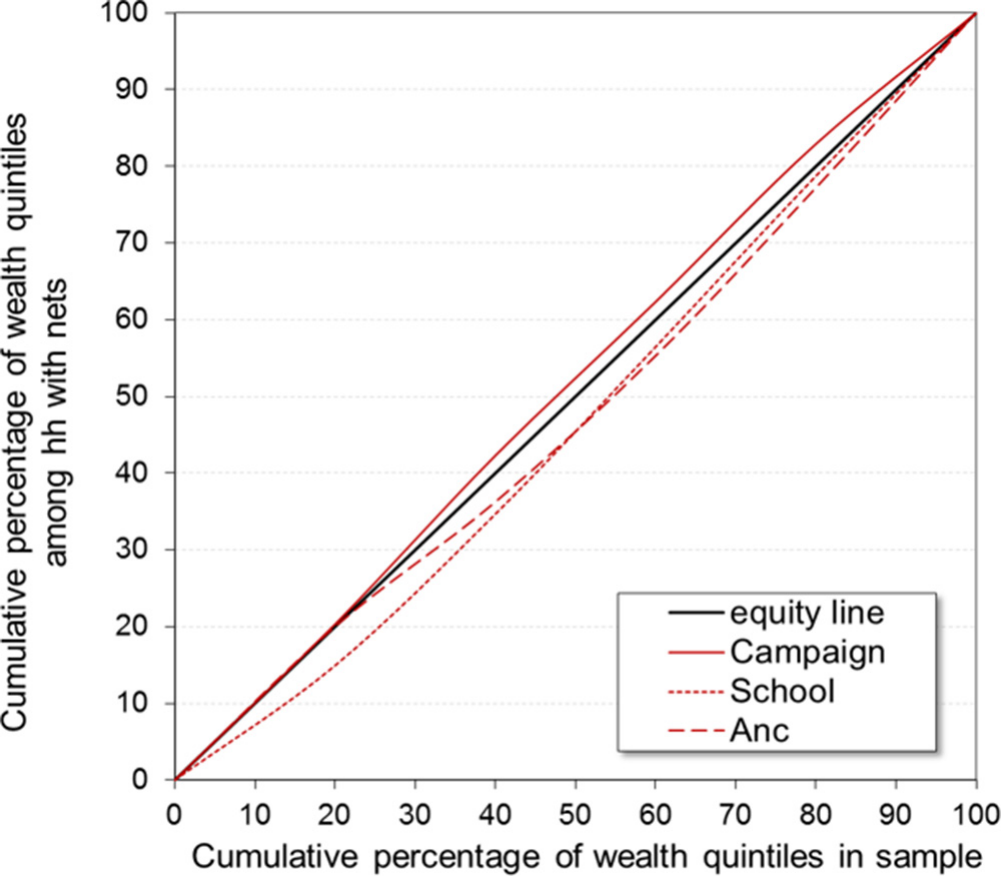
Lorenz Concentration Curve Assessing Equity in Household ITN Ownership by Source of Net Abbreviations: Anc, antenatal care; hh, household; ITN, insecticide-treated net.

Equity of the public distribution channels at the endline survey is shown in Figure 4. Access to campaign ITNs based on recall of the respondent of having received any ITNs from the campaign was highly equitable with a tendency toward a pro-poor distribution (curve above the equity line), but the concentration index of −0.03 (95% CI: −0.08, 0.02) shows that it was not statistically different from perfect equity. School distribution was slightly pro-rich, with a concentration index of 0.06 (95% CI: 0.02, 0.11). Ownership of ANC ITNs was also not statistically different from perfect equity, with a concentration index of 0.04 (95% CI: −0.06, 0.15).

**TABLE 5.** Source of ITNs (%) Among All Households at Endline (N=1,450)

HH Source of ITN	Ikom LGA (Comparison)	Ogoja LGA (2 Rounds)	Obubra LGA (3 Rounds)
**Any source**			
No ITN	56.3 (50.5, 62.0)	22.1 (17.5, 27.6)	20.9 (15.9, 27.0)
At least 1 net from school	0.0	43.0 (35.9, 50.5)	44.2 (35.9, 53.0)
At least 1 net from campaign	31.5 (26.2, 37.3)	18.0 (12.3, 25.6)	29.0 (22.3, 36.8)
At least 1 net from ANC	2.5 (1.4, 4.4)	9.8 (6.9, 13.6)	9.4 (7.1, 12.3)
Other (family, private)	1.9 (0.7, 5.0)	2.5 (1.3, 4.5)	1.7 (0.8, 3.4)
Unknown	8.1 (5.4, 11.9)	11.5 (8.2, 15.8)	6.9 (4.0, 11.6)
**1 Source**			
Campaign only	31.3 (26.0, 37.1)	11.9 (7.2, 19.0)	19.4 (13.8, 26.6)
ANC only	2.3 (1.2, 4.2)	8.4 (5.5, 12.7)	5.6 (4.1, 7.7)
School only	0.0	36.7 (29.9, 44.1)	34.2 (26.4, 43.0)
**2 or more sources**			
Campaign and ANC	0.2 (0.03, 1.5)	0.6 (0.2, 1.9)	1.3 (0.6, 2.6)
Campaign and school	0.0	5.5 (3.6, 8.4)	7.5 (4.9, 11.3)
ANC and school	0.0	0.8 (0.3, 2.1)	1.7 (0.7, 7.7)
Campaign, ANC, and school	0.0	0.0	0.8 (0.3 - 2.1)

Abbreviations: ANC, antenatal care; CI, confidence interval; HH, household; ITN, insecticide-treated net; LGA, local government area.

All data shown as % (95% confidence interval).

### Social and Behavior Change Communication

Endline data on exposure to any net-related messages in the past 6 months based on the recall of household respondents and the sources of those messages are presented in Table 6. The percentage of households reporting exposure to any message varied between the LGAs and was significantly higher in Obubra (51%) and Ogoja (45%) than in the comparison area (32%) (*P*<.001). Radio was the dominant source of messages reported by households in the comparison group at 75% while schools played an increasing role as the duration of school distribution increased (comparison group 4%, Ogoja 17%, Obubra 34%).

**TABLE 6.** Source of Information (%) About ITNs at Endline (N=1,450)

	Ikom LGA (Comparison)	Ogoja LGA (2 Rounds)	Obubra LGA (3 Rounds)
Exposed to information about nets past 6 months	31.5 (24.1, 39.9)	44.5 (37.6, 51.6)	50.7 (45.9, 55.6)
**Source of information if exposed**			
Radio	75.0 (64.6, 83.1)	40.1 (30.9, 50.0)	35.0 (25.8, 45.4)
Health worker (facility or community)	54.6 (46.1, 62.9)	54.4 (45.2, 63.2)	43.2 (33.4, 53.6)
School	4.0 (2.0, 7.6)	17.1 (11.8, 24.0)	33.7 (24.0, 45.1)
Community leader	7.9 (4.5, 13.4)	21.7 (13.7, 32.5)	11.1 (5.6, 20.7)
Town announcer	24.3 (16.1, 35.0)	18.0 (10.0, 30.2)	8.6 (4.9, 14.9)
Family or friends	20.4 (13.5, 29.6)	14.3 (10.0, 20.1)	14.0 (8.0, 23.2)
Pharmacy or shop attendant	10.5 (5.3, 19.8)	0.9 (0.2, 3.5)	0.0
Mosque or church	2.0 (0.6, 6.2)	3.7 (1.8, 7.3)	11.5 (4.7, 25.8)
Newspaper or TV	9.2 (5.3, 15.6)	1.8 (0.7, 4.8)	1.2 (0.3, 5.2)
Mean number of information sources mentioned	2.6 (2.2, 3.0)	2.2 (1.7, 2.7)	1.9 (1.6, 2.2)
Mean number of messages recalled if exposed	3.1 (2.7, 3.5)	2.6 (2.0, 3.3)	3.9 (3.5, 4.3)
Message on net use recalled (all households)	29.0 (21.8, 37.5)	35.0 (29.4, 41.2)	45.3 (39.7, 51.0)
Discussed net use with family	46.8 (40.3, 53.4)	59.0 (53.5, 64.4)	64.1 (57.1, 70.5)
Intention to use nets regularly (most or all nights)	66.1 (59.1, 72.4)	73.4 (67.9, 78.2)	79.3 (74.1, 83.7)
Child learned about malaria and/or nets at school if any schoolchild in household	37.8 (23.1, 55.3)	73.6 (66.5, 79.7)	64.2 (54.1, 73.2)

Abbreviations: ITN, insecticide-treated net; LGA, local government area.

All data shown as % (95% confidence interval).

The most frequently recalled message was “use the net” or “use the net every night,” which was recalled by 85% of respondents reporting exposure to any messages, with higher exposure in the intervention sites and in the site with the longer pilot (comparison group 29%, Ogoja 35%, Obubra 45%; *P*<.001). A similar trend was also seen for discussing net use in the family (*P*=.001) and the intention to use ITNs most or all nights (*P*=.005). Among households with school going children, the proportion whose children had mentioned learning about malaria in class was much higher in the school distribution LGAs (64% to 74%) than the comparison group (38%) (*P*<.05), indicating that schools outside school distribution also discussed or taught about malaria but not as intensively.

## DISCUSSION

Our study found that 3 years after the last mass campaign, ITN ownership and access increased in areas where 2 or 3 rounds of school distribution were implemented. During the same period, ITN ownership and access fell in the comparison area. Oversupply did not significantly increase as a result of the pilot, and school, ANC, and campaign distributions were all very equitable. Very few households obtained ITNs from both ANC and schools, suggesting that the 2 continuous distribution channels have complementary reach. About 40% of all households had an eligible student during the pilot period, and a similar proportion of all households had received a school ITN. Schools were the largest source of ITNs in the intervention LGAs.

Very few households obtained ITNs from both ANC and schools, suggesting they have complementary reach.

At endline, household ownership of at least 1 ITN in the intervention areas was just under the target level of 80% (78% in Obubra and 76% in Ogoja). However, just over half of the population in the intervention areas had access to an ITN. While below the 80% target, population ITN access in the intervention LGAs was within the range observed in post-campaign surveys in other countries.^26^ Although Ogoja (where school distribution started 24 months after the mass campaign) was able to achieve nearly the same coverage rates as the site that started at 15 months post-campaign (Obubra), Obubra benefited immediately from its first round of school distribution, since school ITNs filled some gaps from the mass campaign, which had barely reached half of all households. Ogoja's results, however, show that strong outcomes are possible even when a school distribution program starts late. There may also be a ceiling for ITN ownership and access in a school distribution program with this set of selected grades (4 grades 3 years apart, of which 2 were in secondary school). To reach target levels of population ITN access, future programs may need to use more school grades to reach a broader cross-section of households.

Household ownership of at least 1 ITN was just under the target level of 80% in the intervention areas at endline.

To reach target levels of population ITN access, programs may need to distribute ITNs to more school grades.

Interventions to treat and prevent schistosomiasis and soil-transmitted helminths have long been implemented at schools,^27^ in addition to educational curricula for a wide variety of health interventions,^28^ and malaria programs have implemented intermittent treatment of malaria in school-age children,^29^ school parasitemia surveys,^30^^,^^31^ and school ITN coverage surveys.^32^^,^^33^ WHO recommendations include school distribution of ITNs as part of a comprehensive strategy to maintaining universal coverage, but the most effective combinations of channels are still under study. Other types of distribution (such as through community volunteers and traditional leaders) have been or are being tested in Madagascar,^34^ South Sudan,^35^ and Zanzibar (Mwinyi Khamis, written communication, 2016). An assessment conducted in Tanzania hypothesized that school distribution might be able to replace mass campaigns entirely: A pilot was subsequently conducted, and recent data show that ITN ownership and access were maintained up to 4 years post-campaign. Even households without eligible school children benefited, since 7% of recipient households donated nets to others during the last round of school distribution.^36^ These findings are promising. More work is needed to determine whether these types of programs can maintain ITN coverage over the longer term and whether more consistent coverage year-to-year provides better protection than rising and falling coverage provided by mass campaigns.

Schools offer a practical logistical and administrative platform for ITN distribution. Quantification of ITN needs is based on student enrollment; teachers are by profession literate and able to read and fill out necessary instructions and forms; and school health administration offers a feasible supervision and reporting structure. School distribution is scalable in areas with high school enrollment rates, because it allows countries to leverage existing structures and avoid the time and cost of household registration, and may be particularly useful in places where mass campaigns have been especially challenging. It offers the flexibility to add or subtract grades depending on ITN ownership and access levels achieved.

Equity of school enrollment determines equity of school ITN distribution; less equitable school enrollment rates in Cross River State would likely have resulted in less equitable access to school ITNs. Planners should take enrollment equity into account when planning school distribution programs. Similarly, class selection was based on using primary, junior secondary, and senior secondary school grades to spread out the age ranges of students benefiting from the distribution. Adding or subtracting grades requires more ongoing monitoring than in mass campaigns. Having enough nets was associated with donating nets in the Tanzania study.^36^ Increasing the number of eligible grades could increase levels of sufficient access among school going households and, consequently, redistribution. Furthermore, program planners must weigh the costs of additional transport and training in secondary schools against the benefits of reaching what is usually a smaller number of students (and, consequently, households) in places where secondary school enrollment is low.

As expected, SBCC exposure was much higher in the intervention sites and highest in the site with the longer pilot. Aside from schools themselves, leading sources of information were radio and health workers, indicating that there were malaria SBCC activities beyond the school distribution program. However, more households in the intervention sites had discussed net use and were more likely to have the intention to use nets most nights. Moreover, twice as many households with schoolchildren had a child who learned about malaria in school in the intervention sites than the comparison site. These findings suggest that schools are willing partners in malaria SBCC activities.

### Limitations

This study did have limitations. First, this was not a randomized controlled trial. Though Table 1 suggests that the study groups had similar baseline characteristics, a few slight differences remained that could have influenced the results, such as ownership of a radio, campaign nets, or a means of transport.

Second, while nesting the baseline in a post-campaign survey was necessary to conserve financial resources, the resulting sampling methods for the comparison group varied slightly since the endline was designed solely to assess the coverage achieved by the school distribution pilot, so it was not strictly an intervention-control assessment. While Ikom LGA was merely one in a group of 8 LGAs used in the baseline comparison group, the comparison group at endline comprised households from Ikom LGA only. Table 2 implies it is possible that Ikom LGA was not significantly different from the other LGAs used in the baseline comparison group. However, the baseline Ikom sample size was only 34 (and it was not drawn to be representative of the LGA population as a whole), so this comparison had inadequate statistical power to make any definitive conclusions about comparability.

Third, during the planning stage for school distribution, we assumed that the mass campaign had achieved its targets of 80% of households owning at least 1 ITN, but our baseline survey revealed much lower levels (range, 50.0% to 63.9%). Had this been known earlier, we would have tried to distribute more ITNs through schools during the pilot to make up for the gap. However, this limitation may have been counterbalanced by a longer-than-expected median ITN lifespan. Recent data has shown that median net survival is 4.7 years in Cross Rivers State, much longer than the 3 years we had used in the planning model.^37^

Fourth, the proportion of ITN users among those with access decreased between baseline (rainy season) and endline (dry season). This reflects the potential influence of seasonality (Table 3) on attitudes toward using a net, which has been reported in other net use studies in Nigeria.^38^ It would have been ideal to compare the net use using surveys that had been conducted during the same season. Finally, the study was subject to recall or misclassification bias due to the use of retrospective cross-sectional surveys; families who acquired nets in earlier years may have had more difficulty remembering the source of nets at the time of the surveys.

## CONCLUSION

The addition of school distribution to standard antenatal clinic distribution in Cross River State, Nigeria, increased ownership of at least 1 ITN to nearly 80% and population ITN access to over 50% in the 3 years following a mass campaign; rates fell in the comparison area to 43% and 26%, respectively. School ITN ownership was nearly as equitable as the mass campaign and did not oversupply households. Very few households had ITNs from both school and ANC, indicating that the 2 channels had complementary reach. These results suggest that school and ANC distribution combined can play an effective role in achieving and maintaining universal coverage. Though the proportion of the population with access to an ITN remained similar at baseline and endline, these levels were short of universal coverage. Future programs should consider increasing the number of eligible grades to increase the proportion of the population with access to a net. They should also ensure that ANC distribution programs are functional, to protect biologically vulnerable groups such as pregnant women and infants and contribute to population-level coverage. One of our key learnings is that policy makers should consider school distribution as an option in high school enrollment areas. One size does not need to fit all, and in a vast and very diverse country like Nigeria, having ANC and school distribution in some states and universal coverage campaigns or other forms of continuous distribution programs in other states may be appropriate. More research is underway to evaluate the cost-effectiveness of continuous distribution channels in combination with, or as a potential replacement for, subsequent mass campaigns.

## Supplementary Material

17-00350-Acosta-Supplement.pdf

## Notes

**Peer Reviewed**

**First Published Online:** June 6, 2018
