# Supplementary material for: Design, Implementation, and Evaluation of a School Insecticide-Treated Net Distribution Program in Cross River State, Nigeria
Source: Glob Health Sci Pract. 2018 Jun 27;6(2):272–87. doi: 10.9745/GHSP-D-17-00350 (PMC6024633; doi:10.9745/GHSP-D-17-00350)
Supplement: 17-00350-Acosta-Supplement.pdf [file 17-00350-Acosta-Supplement.pdf]

Acosta A, Obi E, Ato Selby R, et al. Design, implementation, and evaluation of a school insecticide-treated net distribution program in Cross River State, Nigeria. *Glob Health Sci Pract*. 2018;6(2). https://doi.org/10.9745/GHSP-D-17-00350

SUPPLEMENT. Projected ITN Ownership Coverage in Cross River State, Nigeria, From NetCALC

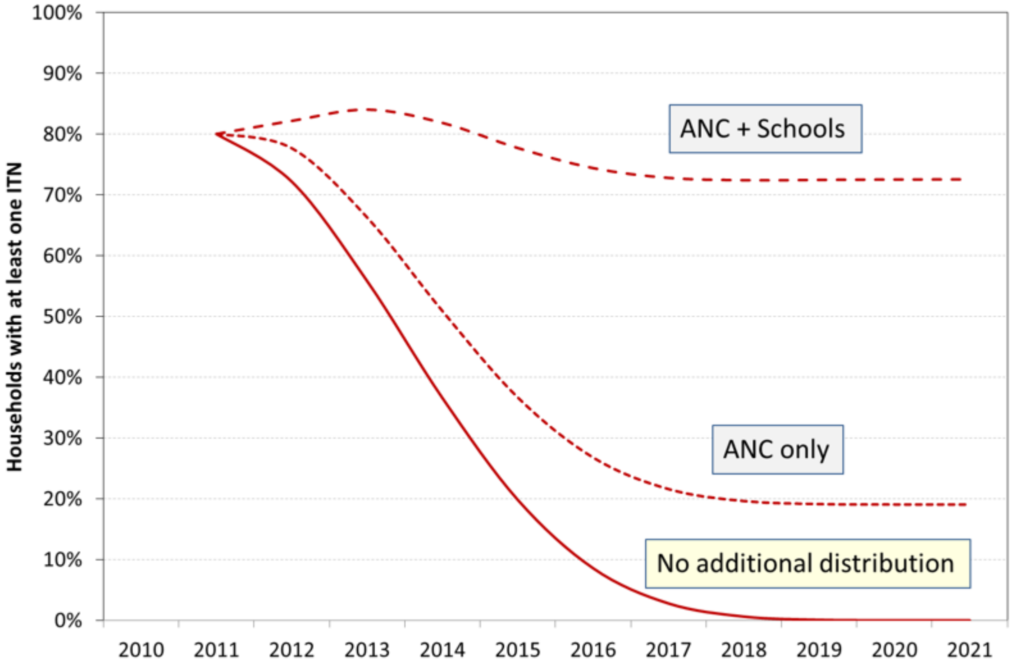

Abbreviations: ANC, antenatal care; ITN, insecticide-treated net.
